# An Exploration of Stress: Leveraging Online Data from Crowdsourcing Platforms

**DOI:** 10.3389/frai.2021.591529

**Published:** 2021-02-18

**Authors:** James Roddy, Samantha Robinson

**Affiliations:** Department of Mathematical Sciences, University of Arkansas, Fayetteville, AR, United States

**Keywords:** Amazon Mechanical Turk, MTurk, TurkPrime, human intelligence tasks, crowdsourcing, perceived stress, differential item functioning, Rasch tree model

## Abstract

**Background:** Early detection of community health risk factors such as stress is of great interest to health policymakers, but representative data collection is often expensive and time-consuming. It is important to investigate the use of alternative means of data collection such as crowdsourcing platforms.

**Methods:** An online sample of Amazon Mechanical Turk (MTurk) workers (N = 500) filled out, for themselves and their child, demographic information and the 10-item Perceived Stress Scale (PSS-10), designed to measure the degree to which situations in one’s life are appraised as stressful. Internal consistency reliability of the PSS-10 was examined via Cronbach’s alpha. Analysis of variance (ANOVA) was utilized to explore trends in the average perceived stress of both adults and their children. Last, Rasch trees were utilized to detect differential item functioning (DIF) in the set of PSS-10 items.

**Results:** The PSS-10 showed adequate internal consistency reliability (Cronbach’s alpha = 0.73). ANOVA results suggested that stress scores significantly differed by education (*p* = 0.024), employment status (*p* = 0.0004), and social media usage (*p* = 0.015). Rasch trees, a recursive partitioning technique based on the Rasch model, indicated that items on the PSS-10 displayed DIF attributable to physical health for adults and social media usage for children.

**Conclusion:** The key conclusion is that this data collection scheme shows promise, allowing public health officials to examine health risk factors such as perceived stress quickly and cost effectively.

## Introduction

Despite the continued media coverage and public interest in those epidemics that dominate current health policy initiatives (e.g., opioid abuse, obesity, and mental illness), stress is an often-overlooked health risk factor. Stress, having an adverse effect on overall health, has been linked to substance abuse, sleep deprivation, obesity, and depression. Detection of stress in adults and children, prior to the development of certain adverse health effects, could allow policymakers to shape the future health of their communities by providing early access to services and interventions. Consequently, it is imperative to explore trends in the stress of both adults and children.

Early detection of stress as a health risk factor is often difficult due to the cost prohibitive nature of data collection. However, taking note of the proliferation and widespread use of crowdsourced online samples in consumer and social science research recruited from popular platforms such as Amazon Mechanical Turk (MTurk), this study proposes that leveraging of survey data from such platforms (when done carefully) can provide policymakers with cross-sectional snapshots of health risk efficiently and at low cost. Reducing the cost burden of data collection can facilitate timely exploratory risk analysis and assist policymakers in the proposal of targeted, systematic studies, and/or interventions.

Using panel data collected from MTurk via TurkPrime with micro-batching enabled, which is a feature allowing for segmented data collection to increase sample representation, current work explores trends in the stress of both adults and their children.

## Methods

### Purpose of the Study

The purpose of the current study was to explore trends in the perceived stress of both adults and their children utilizing survey data collected quickly and easily via a popular crowdsourcing platform, MTurk. MTurk is utilized often in survey research to collect quality data, quickly and at low cost. Buhrmester et al., (2011) conclude that samples from MTurk are more demographically diverse than typical internet samples, participant recruitment is timely and cost-effective, and the data collected are often as reliable as that which are obtained through traditional sampling methods. While these crowdsourced platforms have transformed survey and experimental research in recent years, they are underutilized in public health and preventative medicine. While one primary aim of this study was to demonstrate the potential for leveraging data from crowdsourced online platforms such as MTurk to provide timely and cost-effective access to quality health-related data, the specific focus of the current study was to capture, assess, and explore nonclinical, perceived stress in the general United States (US) population, including both adults and children.

In order to explore perceived stress in both adults and children, as measured by the 10-item Perceived Stress Scale (PSS-10) and while leveraging online data from MTurk, the following research questions were posed:

(1) Does overall self-reported stress in adults differ by demographics?
(2) Do individual survey items on the PSS-10 function differently for adults and/or their children based upon demographics?

### Participants and Data Collection

A crowdsourced online sample of 500 participants was recruited from Amazon’s Mechanical Turk (MTurk) via TurkPrime, a crowdsourcing platform launched publicly in 1995 (Pittman and Sheehan, 2016). MTurk is a popular source for survey participants in research studies, and such studies are typically institutional review board (IRB) exempt (Paolacci et al., 2010).

The target population of interest for the current study was all US adults with children. The MTurk study was launched to gather responses from 500 participants that met the following inclusion criteria (which can be set with TurkPrime panel features):

(1) United States only,
(2) have at least 1 child ≤ 18 years old, and
(3) MTurk approval rate of 90–100% with ≥ 500 previously approved HITs.

The human intelligence task (HIT) was only visible to individuals that qualified. Consequently, all participants were above the age of 18 years (a requirement of all MTurk workers), currently located in the United States, reported having at least one child, and had an MTurk satisfaction rating of 90 percent or above following the advice of Goodman and Paolacci (2017). Participants completed a demographic questionnaire, the PSS-10 for themselves, and an altered PSS-10 based upon their impression of the stress exhibited by one of their children.

A few demographic questions are added to the HIT by TurkPrime allowing a researcher to check (in aggregate) if participants answered survey demographic questions on that particular HIT in a similar manner to the way they answered preliminary demographic questions asked of all MTurk workers. As the demographic questionnaire within the survey included the same demographic questions with similar response choices as those provided by TurkPrime, a basic check for respondent consistency proved tenable. Attention checks, that is, simple ways to determine if a respondent is paying attention to study instructions and survey questions rather than arbitrarily responding (Oppenheimer et al., 2009) were not specifically included within the survey. However, there was a secret key that workers had to correctly supply in order to receive compensation.

Two layers of prevention were utilized in the current study to reduce the possibility of multiple entries from the same individual. First, though anonymous, MTurk workers are provided a unique identification called an MTurk ID, and participants had to supply their MTurk ID in the current survey. There was no more than one survey response for every unique MTurk ID in the current study. While it is theoretically possible for one individual to have multiple MTurk IDs, the MTurk ID must be linked to a unique credit card, which reduces the possibility of multiple responses from an individual. To further prevent multiple entries from the same individual, while also enhancing the diversity and representativeness of the sample, the current study utilized a TurkPrime feature that blocks duplicate IP addresses for the same HIT. Enabling blocking of duplicate IP addresses for the same HIT prevents multiple Turkers that share the same internet connection from taking the survey.

Micro-batching was enabled in this study. Micro-batch HITs take longer to complete, as the survey is batched into smaller HITs with a time delay between each survey launch. Micro-batching allows for segmented data collection and is used to improve sample representativeness (Litman et al., 2017). In the current study, each micro-batch HIT included nine participants per batch and the time delay between successive micro-batch HITs was system optimized. Both the batch size and the time delay can be customized but increasing the number of participants per batch is more costly.

The study launched in July 2019 with an expected run time of two weeks (i.e., 14 days). Despite enabling micro-batching, which typically slows the survey completion time, this survey was completed in less than 5 days.

The expected survey completion time was 15.0 min. Of those completing the survey, the average completion time was 12.7 min and the median completion time was 8.6 min. Participants received compensation in US dollars commensurate with the expected completion time of the survey.

Survey completion statistics are provided when MTurk studies are launched via TurkPrime. The bounce rate, which is the percentage of MTurk workers that qualified for the study and previewed the assignment but did not actually accept the HIT, was approximately 10%. Among those MTurk workers that accepted the HIT, the completion rate for the survey was nearly 80%. Reporting the completion rate, an indicator of overall data quality, is often required or at least encouraged in survey research (Eysenbach, 2004). MTurk attrition is typically due to workers experiencing unanticipated time constraints, loss of interest, or technical difficulties with the HIT.

The completion rate of approximately 80% in the current study was acceptable, especially given that the survey was an external survey, that is, participants were redirected via a hyperlink to the URL of the survey. External survey links provide more opportunity for technical difficulties to arise and may result in higher attrition rates. However, there are benefits to external surveys. For example, the current study survey was created in Google Forms, which allowed for data to be collected and saved in real time as well as allowing for the data to be available in a structure appropriate for analysis immediately via Google Sheets. Creating an external survey allows the researcher to analyze data in real time and to collect responses in a predefined structure most appropriate for the intended data analysis.

### Questionnaire

The Perceived Stress Scale (PSS) is a self-reported instrument used in psychological research to quantify stress (Cohen et al., 1983). The PSS is widely used because it is relatively short, easy to administer, and has been shown to be a reliable and valid measure of perceived stress. While there are multiple versions of the PSS available, a systematic literature review of the psychometric properties of the PSS found the 10-item scale to be superior to both the 14-item and 4-item versions of the PSS (Lee, 2012). Consequently, the PSS-10 developed by Cohen and Williamson (Cohen, 1988) was utilized in the current study.

The PSS-10 measures perceived stress and is not a measure of clinical stress. However, there are thousands of studies published in the last year alone that show a significant correlation between higher PSS-10 scores and clinically measured outcomes. Moreover, scores on the PSS-10 are correlated with outcomes typically associated with clinical stress such as failure to quit smoking, failure among diabetics to control blood sugar levels, greater worsening of eyesight in age-related macular degeneration, earlier onset of multiple sclerosis symptoms, and more reported colds (Ng and Jeffery, 2003; Leung et al., 2010; Gillani et al., 2011; Gitchel et al., 2011; Dougherty et al., 2017).

The items on the PSS-10 ask respondents questions relating to how often in the past month that they have felt upset, felt nervous, felt angry, felt unable to control important things in their lives, or felt on top of things, among many others. The PSS-10 was originally designed to be a unidimensional instrument. As part of exploratory data analysis, factor analysis revealed a two-factor structure. Many researchers have come to the same conclusion finding two factors, often referred to as “perceived stress” and “perceived lack of control” (Nielsen et al., 2016; Nielsen and Dammeyer, 2019). Despite the potential bi-factor structure of the PSS-10, this instrument has consistently shown test–retest reliability, adequate levels of internal consistency reliability, and a common factor structure across various populations (Lee, 2012).

There are both positively worded and negatively worded items on the PSS-10, requiring responses to negatively worded items to be reverse coded. The items included on the PSS-10 ask respondents to self-report the degree to which life has been appraised as stressful in the past month. For example, respondents are asked how often in the past month they have felt confident in their ability to handle their personal problems and how often they have felt things are going their way. The PSS-10 utilizes a 5-point Likert-type scale, that is, never (0), almost never (1), sometimes (2), fairly often (3), or very often (4).

The 10-item PSS consists of the following questions:

1. How often have you been able to control life’s irritations?
2. How often have you felt that things were going your way?
3. How often have you been angered because of things that were outside of your control?
4. How often have you felt difficulties were piling up so high that you could not overcome them?
5. How often have you been upset because of something that happened unexpectedly?
6. How often have you felt that you were unable to control the important things in your life?
7. How often have you felt nervous and “stressed”?
8. How often have you felt confident about your ability to handle your personal problems?
9. How often have you felt you were on top of things?
10. How often have you found that you could not cope with all the things you had to do?

The survey items above were adjusted slightly for the current study, and an additional 10-item set of questions similar to the PSS-10 was used to assess the parents’ perception about their children’s stress. Along with these two versions of the PSS-10, additional demographic information was collected such as but not limited to geographic location, race, education level, employment status, and social media usage.

### Data Analysis

All data preprocessing and analyses were performed in R (R Core Team 2019).

The collected data were preprocessed, with questions 1, 2, 8, and 9 (i.e., the negatively worded items in relation to feelings of stress) reverse coded. Additionally, participants’ home states were collected into both large region classifications (West, Midwest, Northeast, and South) and small region classifications (Pacific, Mountain, West North Central, East North Central, Middle Atlantic, New England, West South Central, East South Central, and South Atlantic) based on US census divisions.

Differences in overall stress level, as measured by the average response on the full set of PSS-10 items, as well as differences in item functionality for the PSS-10 items were explored. Analysis of variance (ANOVA) was used to test for differences in average perceived stress score across a variety of demographic treatments. Specifically, analyses were performed to assess differences in average overall stress based upon location (both large region and small region), race, education level, employment status, and social media usage.

Rasch trees were used to examine differential item functioning (DIF) across various factors, such as physical health and feelings toward social media, for both adults and their children. Rasch trees are a DIF method based upon the Rasch model and recursive partitioning (Strobl et al., 2015).

The Rasch model, created by Georg Rasch, in its most basic form models the probability of answering an item in the affirmative as a function of the person’s ability and the difficulty of the question. For dichotomous response,

$$P\left(X_{ni}=1\right)=\frac{e^{\beta_{n}-\delta_{i}}}{1+e^{\beta_{n}-\delta_{i}}},$$

where $X_{ni}$ is the result from the $n^{th}$ person for question i, $\beta_{n}$ is the ability of person n, and $\delta_{i}$ is the difficulty of question i. A polytomous version of the Rasch model also exists, for ordinal responses that are more complex than just yes/no or success/failure.

The basic algorithm for detecting DIF using Rasch trees is as follows:

1. Estimate the item parameters jointly for all subjects in the current sample, starting with the full sample.
2. Assess the stability of the item parameters with respect to each available covariate.
3. If there is significant instability, split the sample along the covariate with the strongest instability and at the cut point leading to the highest improvement of the model fit.
4. Repeat Steps 1–3 recursively in the resulting subsamples until there are no more significant instabilities (or the subsample become too small).

While the Rasch measurement model is a unidimensional measurement model, the PSS-10 generally shows a bi-factor structure. However, a meta-analysis of articles using the PSS-10 shows that the Cronbach’s alpha for the instrument is acceptable, typically between 0.60 and 0.85 (Tavakol and Dennick, 2011). Lee (2012) reported that Cronbach’s alpha for the PSS regularly exceeded the standard threshold of acceptability at 0.70 and the current sample data yielded a Cronbach’s alpha of 0.73. A large Cronbach’s alpha does not imply unidimensionality, but it does suggest that the measurement is reliable (Hamon and Mesbah, 2002) and that the instruments approximate an essentially unidimensional set of items. Moreover, while unidimensionality is desirable for scale construction (Hambleton and Swaminathan, 1985), violations of unidimensionality may or may not be problematic for DIF detection purposes (De Ayala, 2009).

## Results

There were 500 respondents but, after removing two incomplete responses, a total of 498 individuals were examined. Comparing a few selected sample demographics (see, Table 1) with the 2010 United States census, the sample appears to be fairly representative of the population with the exception of sex and education. Men appear to be underrepresented in the sample and individuals with higher levels of reported education are overrepresented. This finding is in line with previous research findings suggesting that MTurk workers’ demographic information is relatively comparable to the general population of survey respondents (Goodman and Paolacci, 2017).

**TABLE 1:** Selected Sample Characteristics.

Demographic	N	%
Sex		
Male	169	33.94
Female	324	65.06
Other/Prefer not to say	5	1.00
Race/Ethnicity		
Caucasian	376	75.50
African or African American	58	11.65
Asian, Pacific Islander, or Asian American	41	8.23
Other	23	4.62
Home state		
California	37	7.43
Texas	34	6.83
Florida	31	6.22
New York	29	5.82
Ohio	29	5.82
Parent’s education		
Less than high school diploma	6	1.20
High school diploma or equivalent	103	20.68
Associate’s degree	110	22.09
Bachelor’s degree	195	39.16
Master’s degree	73	14.66
Doctorate or terminal degree	11	2.21

ANOVA methods revealed that neither geographic location nor race had a significant effect on overall stress level. However, parent education level, employment status, and characteristics of social media usage did impact overall stress level in adults. Those with higher levels of education experienced lower levels of perceived stress. Those employed full-time also experienced lower levels of perceived stress. Additionally, those survey respondents that reported minimal yet existent social media usage, that is, “once/day” experienced the lowest levels of perceived stress, as measured by the PSS-10 (see, Table 2).

**TABLE 2:** ANOVA Results.

Parent stress ANOVA	F	*p*-value
Large region	0.33	0.801
Small region	0.85	0.562
Race	0.46	0.804
Parent’s education	2.61	0.024*
Parent’s employment status	3.94	0.000*
Parent’s social media usage	2.85	0.015*
**Significant differences by education**	**Difference**	*p* **-value**
Master’s–less than high school	−0.62	0.019*
Master’s–high school	−0.35	0.027*
Master’s–associate’s	−0.23	0.035*
**Significant differences by employment**	**Difference**	*p*-**value**
Full-time–homemaker	−0.28	0.020*
Full-time–unemployed	−0.79	0.048*
Unemployed–prefer not to say	2.37	0.045*
**Significant differences by social media usage**	**Difference**	*p*-**value**
Several times/day–once/day	0.25	0.030*
Several times/week–once/day	0.34	0.014*
Never–once/day	0.71	0.019*

**FIGURE 1:**
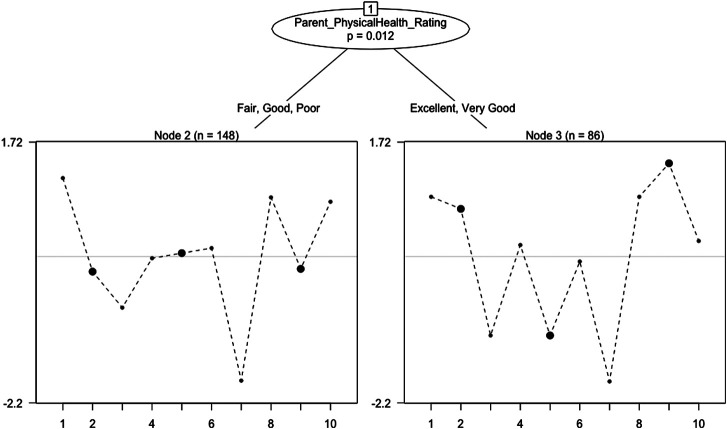
Rasch Tree for PSS‐10 in Adults.

Rasch trees provided evidence that individual items on the PSS-10 functioned differently for adults based upon a self-reported rating of physical health (see, Figure 1).

**FIGURE 2:**
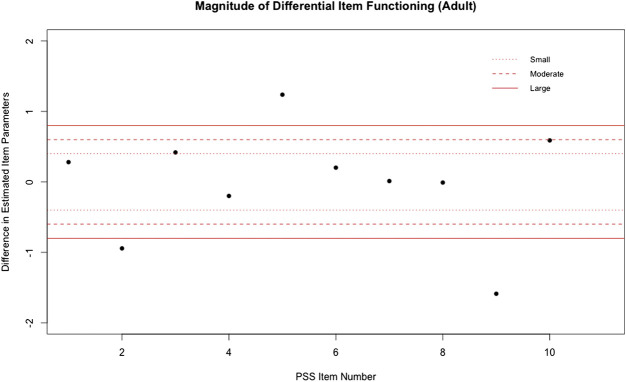
Magnitude of DIF for PSS‐10 in Adults.

Three items (i.e., items 2, 5, and 9) exhibited a large magnitude of uniform DIF, as measured by a difference in estimated item difficulty parameters (see, Figure 2). Specifically, respondents that reported being in excellent or very good health were more likely to feel life was going well than those respondents that reported being in fair, good, or poor health when matched at the same level of overall perceived stress. The group that self-reported being in better health (i.e., those that reported being in excellent or very good health) were also more likely to feel like things were going well for them. However, this better health group appeared to be more likely to be upset by something unexpected happening, when matched at the same level of overall perceived stress.

**FIGURE 3:**
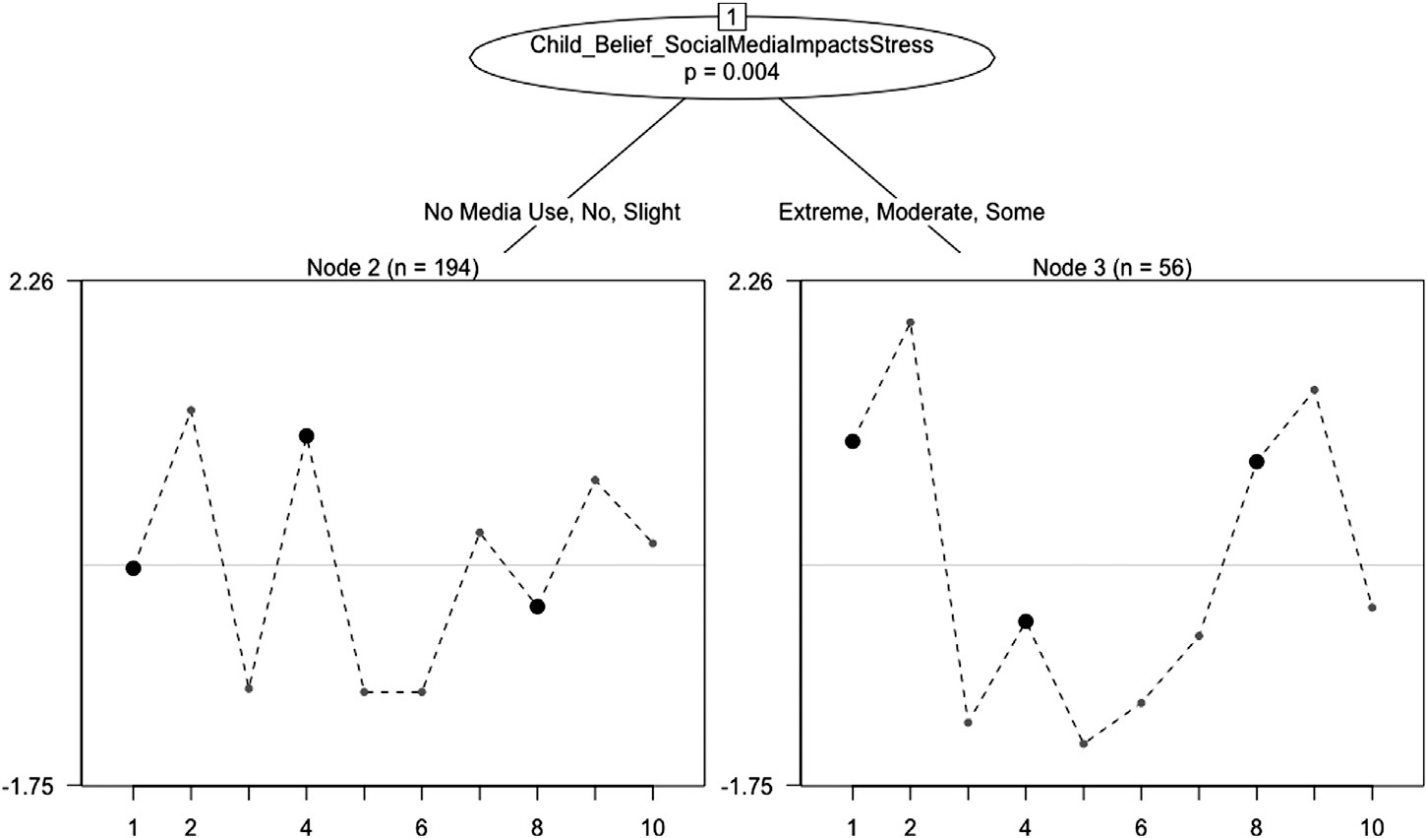
Rasch Tree for PSS‐10 in Children Observed by Adult Respondents.

Rasch trees provided evidence that individual items on the PSS-10 functioned differently when adults answered about their child based upon a self-reported belief that social media impacts stress (see, Figure 3).

Three items (i.e., items 1, 4, and 8) exhibited a large magnitude of uniform DIF, as measured by a difference in estimated item difficulty parameters. Specifically, respondents that reported social media having no or only slight impact on stress less likely to believe that their child could control life’s irritations and were also less likely to believe that their child appeared confident in handling personal problems, when matched at the same level of overall perceived stress for the child. However, these respondents also were less likely to feel that their child had difficulties piling up.

## Conclusion

Those with a master’s degree showed significantly less average stress than those who earned an associate degree or below. Those who are employed full-time had significantly less average stress than those who were homemakers or unemployed. Those who used social media several times per day or per week had higher average stress than those who use it only once per day. Surprisingly, adults who never used social media also reported experiencing higher levels of stress than those who use social media once per day. This possibly shows that periodically using social media to stay connected with people is a good way to relieve stress, while using it too much actually just adds stress. Those with better health, while more likely to feel stress regarding unexpected events, are also more likely to feel things (and life) are going well for them than the lower rated health group when matched on overall self-stress level. Those who believe social media has little impact on stress and those that do not have children that use social media were less likely to feel that their child had difficulties piling up that they could not overcome but, also, less likely to feel their child could control life’s irritations and had the ability to handle personal problems when matched at the same level of overall child stress. Surprisingly, those adults that see social media as a large factor into children’s stress appear more likely to give their child credit for controlling irritations and handling personal problems.

The Rasch model assumes the measurement of interest is unidimensional, which is a potential limitation in the current study. While unidimensionality is not always a problem for DIF detection (De Ayala, 2009), the use of a unidimensional model for data that exhibits a two-factor structure is still considered a potential limitation. A large number of articles in the last two years suggest new ways of addressing the issue of uncertain unidimensionality. For example, multidimensional Rasch model and graphical loglinear Rasch models have been developed (Nielsen and Santiago, 2020). Although the unidimensional Rasch model has been consistently applied in practice, as the PSS-10 is considered an essentially unidimensional instrument with adequate internal consistency reliability as measured by Cronbach’s alpha, the multidimensional Rasch model would be more appropriate. Consequently, the development and use of a multidimensional Rasch tree is a natural extension of the current analysis performed and will be pursued in future work.

Additionally, in using a crowdsourcing platform such as MTurk, the subject pool is limited to a specific segment of the population and is not generally representative. Specifically, participants in this study must both have internet access and be willing to accept the HIT. There was an observed 10% bounce rate, suggesting that 10% of MTurk workers that viewed the survey description did not actually decide to attempt the survey. Additionally, it is unknown how many MTurk workers that met the inclusion criteria for the study did not even view the survey description. However, these limitations to the subject pool exist in almost all survey research (Horton et al., 2011).

Moreover, demographics are just one of the multitudes of potential features impacting stress. While the current study focused on demographics during the ANOVA analysis (especially related to regional variations in perceived stress while also considering basic demographics such as sex, race/ethnicity, and education), it would be beneficial in future work to explore socioeconomic factors as well as health factors that might contribute to mental health such as sleep disturbance, physical activity levels, and substance use.

Despite limitations, these preliminary findings reveal features that contribute differentially to stress levels and, in leveraging survey data from a popular crowdsourcing platform, suggest that policymakers could utilize such platforms to generate cross-sectional snapshots of health risk efficiently and at low cost.

## Data Availability

The raw data supporting the conclusions of this article will be made available by the authors, without undue reservation.
